# Driving an Oxidative Phenotype Protects *Myh4* Null Mice From Myofiber Loss During Postnatal Growth

**DOI:** 10.3389/fphys.2021.785151

**Published:** 2022-02-24

**Authors:** Caiyun Zeng, Hao Shi, Laila T. Kirkpatrick, Aymeric Ricome, Sungkwon Park, Jason M. Scheffler, Kevin M. Hannon, Alan L. Grant, David E. Gerrard

**Affiliations:** ^1^Department of Animal Sciences, Purdue University, West Lafayette, IN, United States; ^2^Meat Science and Muscle Biology Research Group, Virginia Tech, Department of Animal and Poultry Sciences, Blacksburg, VA, United States; ^3^Department of Basic Medical Sciences, Purdue University, West Lafayette, IN, United States

**Keywords:** myosin heavy chain, clenbuterol, oxidative metabolism, muscle fiber type, PGC-1α, myostatin, clenbuterol (CB)

## Abstract

Postnatal muscle growth is accompanied by increases in fast fiber type compositions and hypertrophy, raising the possibility that a slow to fast transition may be partially requisite for increases in muscle mass. To test this hypothesis, we ablated the *Myh4* gene, and thus myosin heavy chain IIB protein and corresponding fibers in mice, and examined its consequences on postnatal muscle growth. Wild-type and *Myh4*^–/–^ mice had the same number of muscle fibers at 2 weeks postnatal. However, the *gastrocnemius* muscle lost up to 50% of its fibers between 2 and 4 weeks of age, though stabilizing thereafter. To compensate for the lack of functional IIB fibers, type I, IIA, and IIX(D) fibers increased in prevalence and size. To address whether slowing the slow-to-fast fiber transition process would rescue fiber loss in *Myh4*^–/–^ mice, we stimulated the oxidative program in muscle of *Myh4*^–/–^ mice either by overexpression of PGC-1α, a well-established model for fast-to-slow fiber transition, or by feeding mice AICAR, a potent AMP kinase agonist. Forcing an oxidative metabolism in muscle only partially protected the *gastrocnemius* muscle from loss of fibers in *Myh4*^–/–^ mice. To explore whether traditional means of stimulating muscle hypertrophy could overcome the muscling deficits in postnatal *Myh4*^–/–^ mice, myostatin null mice were bred with *Myh4*^–/–^ mice, or *Myh4*^–/–^ mice were fed the growth promotant clenbuterol. Interestingly, both genetic and pharmacological stimulations had little impact on mice lacking a functional *Myh4* gene suggesting that the existing muscle fibers have maximized its capacity to enlarge to compensate for the lack of its neighboring IIB fibers. Curiously, however, cell signaling events responsible for IIB fiber formation remained intact in the tissue. These findings further show disrupting the slow-to-fast transition of muscle fibers compromises muscle growth postnatally and suggest that type IIB myosin heavy chain expression and its corresponding fiber type may be necessary for fiber maintenance, transition and hypertrophy in mice. The fact that forcing muscle metabolism toward a more oxidative phenotype can partially compensates for the lack of an intact *Myh4* gene provides new avenues for attenuating the loss of fast-twitch fibers in aged or diseased muscles.

## Introduction

Skeletal muscle consists of a diverse set of muscle cells that can be classified by the relative expression of major sarcomere myosin heavy chain (MyHC) isoforms (Schiaffino and Reggiani, 1994; Weiss and Leinwand, 1996; Pette and Staron, 2000), whose ATPase activity is reflective of the contractile speed of the muscle fiber and correlates with the fiber’s metabolic profile (Schiaffino and Reggiani, 1994; Pette and Staron, 2000; Spangenburg and Booth, 2003; Bourdeau Julien et al., 2018). Among the eleven different MyHC isoforms found in mammal skeletal muscle, two are found in developing muscles, i.e., MyHC-embryonic and MyHC-neonatal, coded by *Myh3* and *Myh8* respectively; while the other nine are found in adult muscles (Schiaffino et al., 1989; Baldwin and Haddad, 2002; Schiaffino, 2018). In adult mice, most muscle fibers consist homogeneously of either type of MyHC I, IIA, IIX(D) or IIB isoforms, encoded by *Myh7*, *Myh2*, *Myh1*, and *Myh4*, respectively. Even so, a small number of fibers contain a mixture of isoforms reflecting their ability to transition to neighboring isoforms and fiber types following an obligatory pathway: I ↔ IIa ↔ IIx ↔ IIb (Schiaffino and Reggiani, 1994; Pette and Staron, 2000; Schiaffino, 2018). At birth, adult fast-twitch MyHCs represent less than 1% of the total MyHC in muscle; yet fast isoforms accumulate rapidly during the postnatal period (Allen and Leinwand, 2001) and ultimately consist of a disproportionate majority (> 70%) of the totality of muscle fibers in the musculature of adult mice and other mammals (Hamalainen and Pette, 1993).

Although the ontogeny and functional significance of muscle fiber specification throughout life are well-documented, the impact of fiber type diversity during growth remains limited. Early skeletal muscle development is governed by a myriad of sequentially expressed transcription factors such as MyoD, Myf5, Mrf4, and Myogenin, and their downstream targets (Braun and Gautel, 2011; Brown, 2014). During late fetal development, fiber type specification begins in response to a distinct set of fetal specific transcription factors (Grifone et al., 2004; Niro et al., 2010) and transcriptional repressors (Hagiwara et al., 2007; von Hofsten et al., 2008). Immediately after birth, substantial muscle growth and cellular hypertrophy accompany fiber type transition from oxidative to more glycolytic adult fiber types, a process most likely triggered by innervation-mediated, calcium-orchestrated signaling cascades (Olson and Williams, 2000; Serrano et al., 2001; Spletter and Schnorrer, 2014). Although neuronal activity drives fiber type specification, metabolite-dependent signaling, such as AMPK and mTORC1, and transcription factors, such as PPAR and PGC-1α, also help fine tune fiber type specification (Bourdeau Julien et al., 2018). Recent studies provide more evidence to support the notion that metabolites can drive muscle fiber switching through AMPK-PGC1α axis. For examples, quercetin, found in many fruits, vegetables and grains, can induce muscle fiber switching to a more oxidative phenotype through adiponectin-AMPK-PGC-1α pathway (Chen et al., 2021). Dietary supplementation of arginine to mouse induces fast- to slow-twitch fiber type transition, accompanied by an upregulation of Sirt1/AMPK pathway (Chen et al., 2018). Other studies also show that nutrients can promote slow fiber program via AMPK signaling (Wen et al., 2020; Xu et al., 2020a,b).

Associated with fiber type specification after birth, muscle fibers experience tremendous hypertrophy, especially in fast-twitch fibers. This fiber hypertrophy is critical for muscle accumulation postnatally, because most reports show that muscle fiber number is set at birth (Rowe and Goldspink, 1969), although this is still somewhat controversial. For example, Li et al. (2015) show that although certain muscles such as gastrocnemius and longissimus dorsi contain fibers determined prenatally, other muscle groups such as TA and EDL continue to increase in fiber number within the first week of life. Compared to slow-twitch, oxidative muscle fibers, fast-twitch, glycolytic fibers are larger in size and tend to experience greater hypertrophy in response to growth stimuli such as clenbuterol (Shi et al., 2007), suggesting fiber type-based hypertrophy may be responsible for overall lean accretion in muscle. Consistent with this notion, mean body mass is reduced by 27 and 18% in MyHC IIX(D)- and IIB- knockout mice, respectively, at 6 weeks of age, compared to wild-type mice (Acakpo-Satchivi et al., 1997). In these mice, the percentages and cross sectional areas of the remaining fiber type increased, however, not to a level sufficient to rescue the normal muscle phenotype (Allen et al., 2001; Allen and Leinwand, 2001), supporting the thesis that fast-twitch fibers, particularly IIX(D) and IIB fibers are necessary for optimal skeletal muscle growth. In this study, we used *Myh4*^–/–^ mice to explore the hypertrophic potentiality of the existing fiber types. Most importantly, we show that by pushing muscle metabolism to oxidative phenotype can partially rescues the loss of muscle mass and fiber number caused by the lack of functional IIB fibers.

## Materials and Methods

### Animals

All animal procedures were approved by the Purdue University Animal Care and Use Committee. The wild-type and genetically mutant mice were from C57BL/6 background. MyHC-IIB knockout (*Myh4*^–/–^) mice were kindly provided by Dr. Leslie A. Leinwand (University of Colorado, Boulder, Colorado). PGC-1α transgenic (*Ppargc1a*^Tg/+^) mice were a kind gift from Bruce M. Spiegelman (Harvard University). Myostatin knockout (*Mstn^–/–^*) mice provided by Se-Jin Lee (Johns Hopkins University). PGC-1α transgenic mice and myostatin knockout mice were crossed with MyHC-IIB knockout mice to generate double knockout mice. For clenbuterol treatment, 20 parts per million (ppm) clenbuterol (Sigma, St. Louis, MO) in drinking water was fed to mice *ad libitum* for 2 weeks. For 5-aminoimidazole-4-carboxamide 1-β-D-ribofuranoside (AICAR, Sigma, St. Louis, MO) study, AICAR was first injected subcutaneously into the mother of the newborn mice for 3 weeks, then directly into the male pups after 3 weeks. The dosage for AICAR was 500 μg per gram body weight for the first week, and 1,000 μg per gram body weight for week 2 and 3. For weeks 4–6, the dosage is 500 μg per g body weight. For each genotype and treatment, six male mice were used for data collection and statistical analysis. To reduce long-term genetic variations, littermates were used for all experiments outlined herein. Specifically, when *Myh4*^+/–^ males and females were mated, *Myh^+/+^* and *Myh4*^–/–^ mice each were observed at 25%. These mice were then used as wild-type (WT) and knockouts (KO) in all experiments.

### Immunohistochemical Staining of Muscle Fiber Types on Serial Sections

Serial sections from the muscle mid-belly were cut 10 μm thick using a cryomicrotome (Walldorf, Germany) and placed on silane-coated slides. Prior to immunohistochemistry, slides were removed from a –80°C freezer and air dried at room temperature (RT) for 1 h. Slides were blocked for 1 h at RT in phosphate buffered saline (PBS) containing 5% goat serum (blocking buffer). After three 5 min washes in PBST (0.1% Tween 20), serial sections were incubated at 37°C for 1 h with primary antibodies against MyHC-I (A4.840), IIA (SC-71), or IIB (BF-F3) (Developmental Study Hybridoma Bank, Iowa City, IA) diluted at 1:100 in the blocking buffer. To detect IIX fibers, we applied all three antibodies to muscle sections, unstained muscle fibers were identified as pure IIX fibers. After three additional 5 min washes in PBST, sections were incubated at 37°C for 1 h with goat anti mouse IgG and IgM secondary antibody (Jackson ImmunoResearch, 115-065-044, West Grove, PA) diluted 1:1,000 in the blocking buffer. After a 3 × 5 min wash in PBST, sections were incubated at 37°C for 45 min with peroxidase-streptavidin (Jackson ImmunoResearch, 016-030-084, West Grove, PA) diluted 1:1,000 in the blocking buffer. After a 3 × 5 min wash in PBST, MyHCs were visualized using a DAB reaction kit (Vector Laboratories, SK-4100, Burlingame, CA).

### Measurement of Muscle Fiber Size, Total Fiber Number, and Fiber Type Percentage

Cross sectional area (μm^2^) of muscle fibers and selected fields on muscle sections were measured using the Adobe Photoshop or NIH ImageJ software. A micrometer image taken at the same magnitude as muscle sections was used as a reference to convert pixel into μm^2^.

To count the total number of the muscle fiber, low-magnitude (40x) images of muscle sections were taken and analyzed using the NIH ImageJ software. For TA and EDL muscle, we counted all the fibers in the muscle. For gastrocnemius muscle, we selected 10 random fields from the whole gastrocnemius muscle with each area containing 200–300 fibers. We then measured the cross-sectional area (μm^2^) of the selected field using a micrometer image taken at the same magnitude as muscle sections as a reference to convert pixel into μm^2^. We then counted the total number of fibers within that field to obtain the fiber/μm^2^ ratio. The area of the whole gastrocnemius muscle was calculated using a micrometer as a reference. Low magnitude images (40x or 10x) of the whole gastrocnemius muscle section along with a micrometer were taken and analyzed using NIH ImageJ as described above. By multiplying the fiber/μm^2^ ratio with the total μm^2^ of the gastrocnemius muscle, we obtained an estimation of the total number of fibers per gastrocnemius. To assess the accuracy of our estimation model, we counted the total numbers of 10 wild-type gastrocnemius muscles and found this estimation approximated the actual count with a percent error of 0.5%. For *Myh4*^–/–^ knockout, we counted all the fibers.

To measure the percentage of Type I, IIA, IIX, and IIB fibers in the gastrocnemius muscle, we counted the total number of each fiber type and divided this number by the sum of the number of all four types.

### Adult Skeletal Muscle Electroporation

An *Myh4*-luciferase reporter containing *Myh4* gene sequence from –2,560 to + 13 bp was kindly provided by Dr. Steven J. Swoap (Williams College, Williamstown, MA, United States) and described elsewhere (Swoap, 1998; Swoap et al., 2000). Plasmid pRL-SV40 (Promega, Madison, WI) encoding Renilla luciferase, was co-injected with the reporter to normalize transfection efficiency. Plasmids were purified using an EndoFree plasmid mega kit (Qiagen, Valencia, CA) diluted to a final concentration of 0.5 μg/μl in sterile 0.45% NaCl. Electroporation was performed as follows: two spatula electrodes were placed on each side of the muscle belly, and eight pulses (1 Hz, 200 v/cm) at 20 ms intervals were applied using a BTX ECM electroporator (Genetronics, San Diego, CA). Two weeks after electroporation, gastrocnemius muscles were collected and homogenized in passive lysis buffer (Promega), and luciferase activity was measured in a TD-20/20 luminometer (Turner Designs, Sunnyvale, CA) using a Dual-Luciferase assay kit (Promega).

### Immunoblotting

Gastrocnemius muscles were snap frozen in liquid nitrogen and homogenized in ice-cold RIPA lysis buffer (1% Nonidet P-40, 0.1% SDS in 50 mM NaCl, and 20 mM Tris, pH 7.6) in the presence of 1 mM PMSF, aprotinin (10 μg/ml), leupeptin (10 μg/ml), 50 mM NaF, and 1 mM Na_3_VO_4_. Homogenates were centrifuged at 13,000 × *g* at 4°C for 10 min, and supernatants were analyzed for protein concentration with the Bio-Rad Protein Assay Kit II (Bio-Rad, #5000002, Hercules, CA). An aliquot (100 μg) was separated using SDS-PAGE (10%). Proteins were transferred to PVDF membranes, which were blocked in 5% non-fat milk at 4°C overnight. After three 5 min washes, membrane was incubated with primary antibodies followed by secondary antibody incubation. Where indicated, blots were stripped and re-probed with appropriate antibodies. Primary antibodies against ERK1/2 (#4696), P38 (#9212), and JNK (#9252) and their respective phosphorylated forms pERK1/2 (#4376, T202/Y204), pP38 (#9216, T180/Y182), and pJNK (#9255, T183/Y185) were purchased from Cell Signaling Technology (Danvers, MA). Antibody-antigen complexes were visualized using an ECL kit (Amersham, Piscataway, NJ).

### Data Analysis

Numerical data were graphed using Microsoft Excel software. Data are reported as mean ± standard error of mean (SEM). Statistical analysis was performed using the mixed model of JMP (SAS Institute, Cary, NC). Body weight, muscle weight, total fiber number, cross sectional area, fiber type percentage, and densitometry of Western blots were compared between genotypes and treatments. Asterisks or letters that differ indicate significance level at *P* < 0.05.

## Results

### Ablation of *Myh4* Reduces Muscle Mass Accumulation Postnatally

**FIGURE 1:**
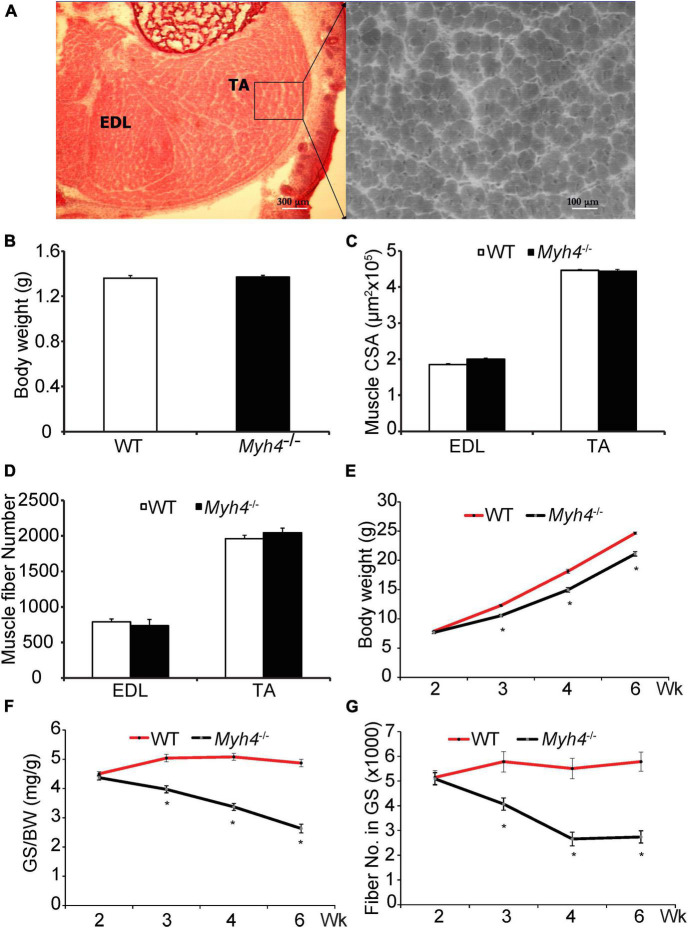
Mice lacking MyHC-IIB exhibit reduced body weight, muscle weight, and fiber number 2 weeks postnatally. **(A–D)** Newborn (day 1) muscle cross section **(A)**, body weight **(B)**, muscle cross sectional area **(C)** and fiber number **(D)** of *extensor digitorum longum* (EDL) and *tibialias anterior* (TA) were measured. Body weights **(E)**, weights of the *gastrocnemius* (GS, **F**), and total muscle fiber number **(G)** were recorded for WT and *Myh4*^–/–^ mice at 2, 3, 4, and 6 weeks of age. Data represent means ± SEM, *n* = 6 in each genotype. Asterisks and letters that differ indicate significance (*P* < 0.05).

Mice lacking functional MyHC-IIB fibers have reduced muscle mass (Acakpo-Satchivi et al., 1997; Allen and Leinwand, 2001; Desgeorges et al., 2017), however, it is unknown whether the loss of muscle mass happens *in utero* or postnatally. We found that the body weights of newborn wild-type and *Myh4* knockout mice were comparable, at approximately 1.3 g (*P* > 0.05; Figure 1B). We then measured the cross-sectional area (Figures 1A,C) and the total fiber number (Figures 1A,D) of the EDL and TA from wild-type and *Myh4* knockout mice. Similar to weight, no differences in these muscle parameters were detected across genotypes (*P* > 0.05). These data suggest loss of the ability to generate MyHC-IIB fibers had no effect on muscle mass development suggesting loss of muscle mass in *Myh4* null mice occurs postnatally. To narrow down the time window of muscle loss postnatally in *Myh4* knockout mice, we measured the body weight (Figure 1E), muscle weight (Figure 1F), and muscle fiber number (Figure 1G) of gastrocnemius (GS) muscle 2, 3, 4, and 6 weeks postnatally. We chose gastrocnemius muscle because MyHC-IIB fibers are the most predominant fiber type in that muscle, and as such, knockout *Myh4*, and thus MyHC-IIB fibers would have a dramatic influence on the GS. The body weight of *Myh4* null mice were significantly lower than that of the wild-type mice starting at week 3 of age (Figure 1E). *Myh4*^–/–^ gastrocnemius muscle weight followed the same pattern as the body weight (Figure 1F). Interestingly, the reduction of muscle fiber number in gastrocnemius muscle occurred between week 2 and 4, stabilizing after week 4, although between week 4 and 6, muscle weight continued declining. Together, these data suggest the loss of MyHC-IIB fibers reduces the capacity of muscle to increase in size and this effect follows a tight temporal pattern postnatally.

### Muscle Partially Compensates for Loss of Type IIB Fibers

**FIGURE 2:**
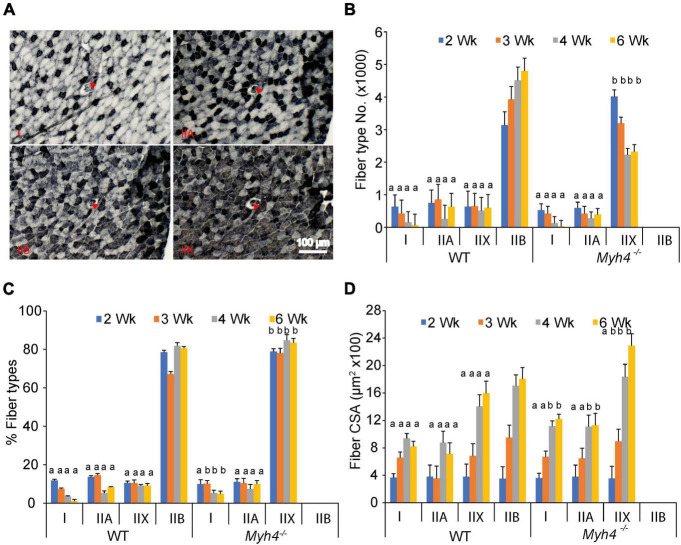
MyHC-IIB deficient muscles have altered muscle fiber composition and fiber size. **(A)** Representative images of serial muscle sections stained with anti-myosin heavy chain I, IIA, IIB and all three antibodies (IIX, white non-stained). Red arrows point to the same fiber across the serial sections. Number (X 1000) of each adult fiber type **(B)**, percent fiber type composition **(C)**, and fiber cross sectional area **(D)** were counted in the *gastrocnemius* of 2, 3, 4, and 6 week-old mice. Data represent means ± SEM, *n* = 6 in each genotype. Means bearing different letters differ (*P* < 0.05) in the same fiber types at the same time point between wild-type and knockout mice.

To further dissect the causes of muscle loss in *Myh4* knockout mice, we examined muscle fiber type composition and the cross-sectional area of each fiber in the gastrocnemius muscle. We stained serial muscle sections with anti-myosin heavy chain I, IIA, and IIB antibodies to identify type I, IIA and IIB fibers (Figure 2A). For IIX fibers, we stained the section with all three myosin heavy chain antibodies and counted the non-stained fibers as IIX fibers (Figure 2A). In wild-type mice, the percentage of type I fibers in the GS muscle gradually decreased in frequency between 2 and 6 weeks. Type IIA and X(D) fibers remaining relatively constant over the same timeframe (Figure 2B). Conversely, IIX(D) fibers predominated the GS muscle in *Myh4* null mice over the same period (Figure 2B). In the GS muscle of wild-type mice type IIB was the dominate fiber type, accounting for some 80% of all fibers postnatally (Figure 2C). In contract, type IIX(D) accounted for more than 80% of fibers in null mice postnatally (Figure 2C). Common across both wild-type and knockout gastrocnemius muscles was the observation that the percentage of type I fibers continuously decreased consistently between week 2 and 6. We then examined the cross-sectional area of each fiber types in wild-type and *Myh4* knockout mice. In wild-type mice, all the four fiber types increased postnatally, reaching a plateau approximately 4 weeks postnatally (Figure 2D). Whereas in *Myh4* knockout gastrocnemius, type I and IIA reached a plateau at week 4, type IIX(D) fibers continued to enlarge through 6 weeks of age (Figure 2D). Together, these findings show that muscle attempts to compensate for the lack of functional type IIB fibers by increasing the size and frequency type IIX(D) fibers as well as increasing the sizes of the remaining fiber types.

### Shifting Muscle Metabolism to Oxidative Phenotype Partially Rescues Muscle Loss Caused by *Myh4* Ablation

**FIGURE 3:**
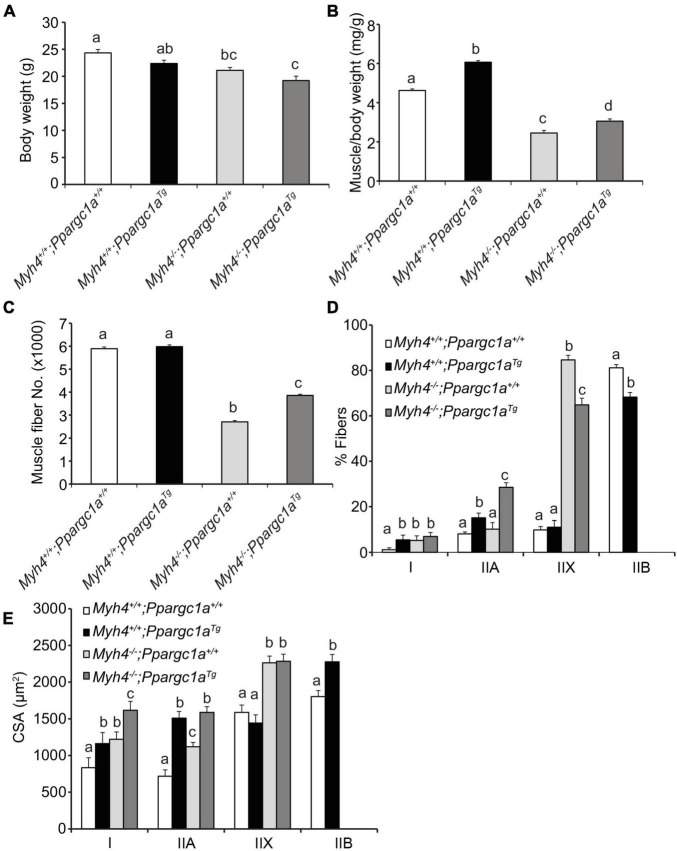
Over-expression of PGC-1α partially rescues muscle mass and fiber number in MyHC-IIB deficient mice. *Ppargc1a* transgenic mice were crossed with WT and *Myh4*^–/–^ mice. Body weight **(A)**, *gastrocnemius* (GS) weight **(B)**, fiber number **(C)**, composition (%) of muscle fiber types **(D)**, and cross-sectional area (CSA) of each fiber type **(E)** were recorded. Mice were 6-week-old males. Data represent means ± SEM, *n* = 6 in each genotype. Means bearing different letters differ (*P* < 0.05).

Preliminary data showed that after a mouse is born, its muscle fiber shifts from type I, oxidative type to type IIB, glycolytic types within the first 4 weeks. As such, we hypothesized that as muscle fibers attempt and fail to transition to the type IIB phenotype, fibers may be lost because the slower contracting programs are blunted. In an attempt to address this possibility and mitigate the loss in fibers, we crossed *Myh4*^–/–^ mice with *Ppargc1*^*Tg*/+^ mice to generate double mutant mice. We chose *Ppargc1*^*Tg*/+^ mice because they exhibit a fast-to-slow fiber type switching when PGC-1α is overexpressed in skeletal muscle (Lin et al., 2002); on the other hand, knockout of PGC-1α promotes fiber type switching from oxidative type I and IIA fibers to glycolytic type IIX and IIB fibers (Handschin et al., 2007). We found the body weight of *Ppargc1a*^Tg/+^ mice were slightly lower than that of wild-type mice, though not at a significant level (Figure 3A). On the other hand, *Ppargc1a* transgenics increased the relative gastrocnemius mass (muscle weight/BW) in both *Ppargc1a*^Tg/+^ and double mutant mice (Figure 3B). Although *Ppargc1a*^Tg/+^ mice did not exhibit increased muscle fiber number in gastrocnemius, it partially rescued gastrocnemius fiber number in double mutant mice (Figure 3C). We then examined the percentage of each fiber type in gastrocnemius and found that *Ppargc1a*^Tg/+^ muscle had reduced type IIB fibers and correspondingly increased oxidative type I and IIA fibers (Figure 3D). Overexpressing PGC-1α greatly reduced IIX fibers and increased IIA fibers in the MyHC-IIB deficient gastrocnemius (Figure 3D). Additionally, *Ppargc1a*^Tg/+^ transgenics significantly increased type I and IIA fiber cross sectional area in both *Ppargc1a*^Tg/+^ and *Ppargc1a*^Tg/+^/*Myh4*^–/–^ mice (Figure 3E). Together, these data show that shifting muscle metabolism and presumably its signaling from glycolytic to oxidative phenotype partially rescued muscle fiber and mass loss caused by *Myh4* ablation.

### AICAR Treatment Mimics PGC-1α Overexpression Effect

**FIGURE 4:**
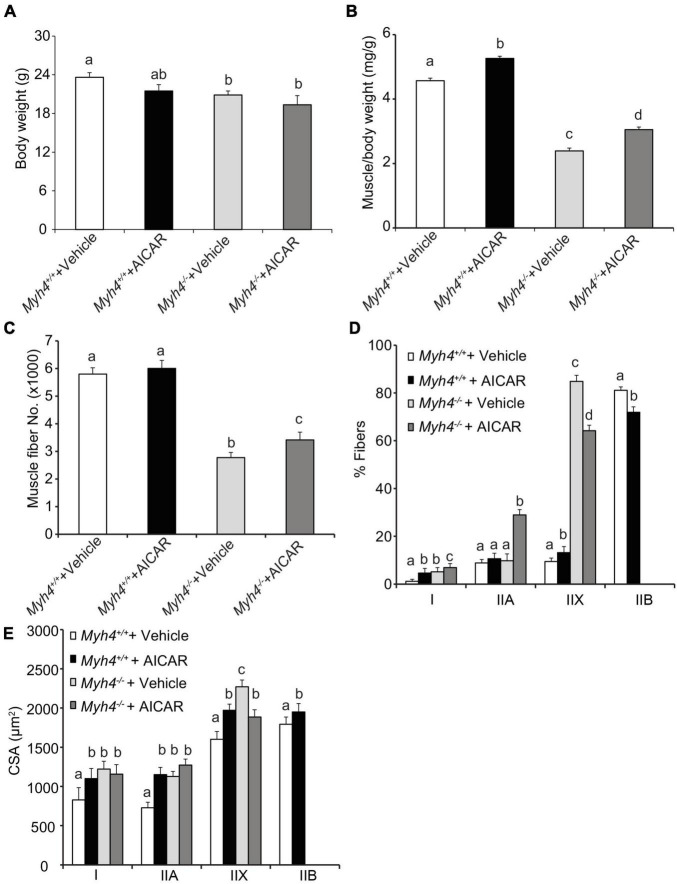
AICAR treatment partially rescues muscle mass and fiber number in mice lacking a functioning *Myh4* gene. Mice were treated with AICAR for 6 weeks. Body weight **(A)**, *gastrocnemius* weight **(B)**, fiber number **(C)**, muscle fiber type composition **(D)**, and cross-sectional area (CSA) of each fiber type **(E)** were recorded. Mice were 6-week-old males. Data represent means ± SEM, *n* = 6 males in each genotype. Means bearing different letters differ (*P* < 0.05).

To confirm our transgene efforts, we used 5′-aminoimidazole-4-carboxamide 1-β-D-ribofuranoside (AICAR), a widely used compound to shift muscle metabolism to a more oxidative phenotype, to examine whether we could replicate the PGC-1α effect. AICAR is a pharmacological nucleoside, once transported into cells, it is metabolized to 5′-aminoimidazole-4-carboxamide 1-β-D-ribofuranosyl monophosphate (ZMP). Like endogenous AMP, ZMP is a potent AMP-activated kinase (AMPK) agonist (Ljubicic and Jasmin, 2013). Once activated, AMPK functions through silent mating-type information regulator 2 homolog 1 (SIRT1)-mediated PGC-1α activation to promote mitochondrial biogenesis (Canto et al., 2010; Viollet, 2017). Additionally, chronic AICAR treatment has been shown to significantly reduce type IIB fibers (Desgeorges et al., 2017), yet increases IIX fibers in rat extensor digitorum longus (EDL) muscle (Suwa et al., 2003). We administrated AICAR for 6 weeks, first through maternal milk, then directly into the weaned pups, and found AICAR had similar effects on body weight (Figure 4A), and gastrocnemius weight (Figure 4B), fiber number (Figure 4C), percent fiber type composition (Figure 4D), and fiber size (Figure 4E), as *Ppargc1a*^Tg/+^ transgenics, though to a different extent. These data suggest that although *Ppargc1a*^Tg/+^ transgenics and AICAR may function through different mechanisms, shifting muscle metabolism signaling to a more oxidative phenotype helps rescue or overcome some of muscle fiber losses in MyHC-IIB deficient mice.

### Knockout of Myostatin Fails to Rescue Muscle Loss Caused by *Myh4* Ablation

**FIGURE 5:**
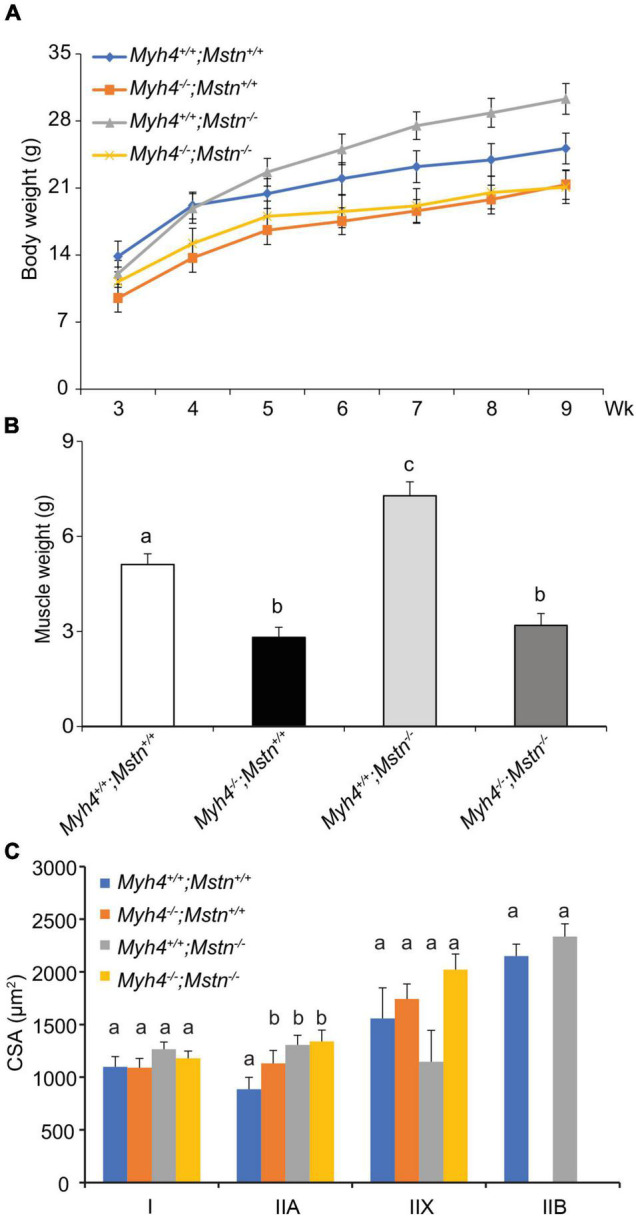
Lack of myostatin fails to rescue muscle growth deficiencies in MyHC-IIB null mice. *Myh4*^–/–^ mice were crossed with *Mstn^–/–^* mice to generate double knockout mice. Body weight of each genotype was recorded from 3 to 9 weeks **(A)**. By the end of week 9, *gastrocnemius* (GS) weights **(B)** and the cross-sectional area of each muscle fiber type **(C)** were assessed. Data represent means ± SEM, *n* = 6 males in each genotype. Means bearing different letters differ (*P* < 0.05).

By pushing muscle metabolism toward more oxidative phenotype, we showed that muscle mass and fiber loss can be partially rescued. Next, we asked whether IIB fibers are required for maximal muscle hypertrophy. In other words, could the existing muscle physiology created by the loss of *Myh4*^–/–^ be stimulated to compensate for the loss of muscle mass and fiber number in these mice. To answer this, we chose the well-studied “double muscling” mice lacking myostatin (*Mstn^–/–^*) and crossed them with *Myh4*^–/–^ mice to generate double knockout mice (*Mstn^–/–^*; *Myh4*^–/–^). Inactivation of myostatin in mice (McPherron et al., 1997) cattle (Grobet et al., 1997; Kambadur et al., 1997; McPherron and Lee, 1997), humans (Schuelke et al., 2004), sheep (Clop et al., 2006), dogs (Mosher et al., 2007), as well as horses (Bower et al., 2012; Petersen et al., 2013), dramatically increases muscle mass and/or muscle strength/performance. In addition, *Mstn^–/–^* mice also show increased total fiber number and contractility properties of muscles (Mendias et al., 2006). *Mstn^–/–^* mice had significantly heavier body weights than the wild-type mice, however, lack of myostatin failed to rescue the lowered body weights of *Myh4*^–/–^ mice (Figure 5A). Similarly, *Mstn^–/–^* gastrocnemius exhibited profound hypertrophy (Figure 5B), however, myostatin deficiency failed to rescue muscle loss caused by MyHC-IIB deficiency (Figure 5B). Moreover, the loss of myostatin was incapable of increasing the cross-sectional area of any of the fiber types in MyHC-IIB deficient gastrocnemius (Figure 5C), raising the possibility that part of the increases in muscularity noted in myostatin null animals may be related to shifts in muscle fiber composition.

### Mice Lacking *Myh4* Fails to Respond to Clenbuterol-Induced Adult Muscle Hypertrophy

**FIGURE 6:**
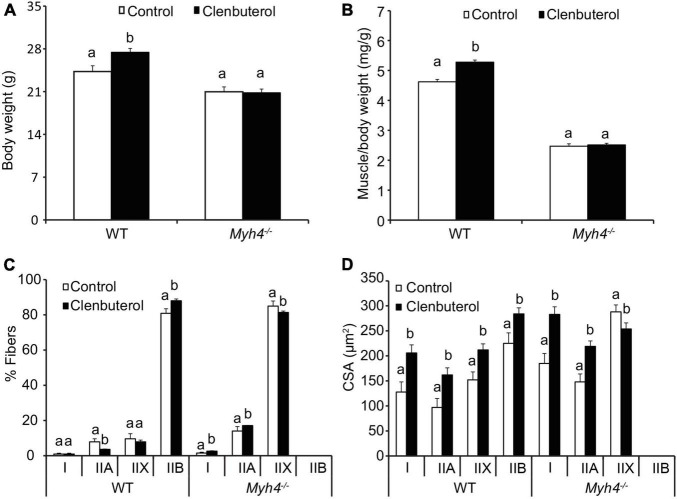
Mice lacking *Myh4* fails to respond to clenbuterol-mediated muscle hypertrophy. Four-week-old male mice were fed 20 ppm clenbuterol in drinking water for 2 weeks, body weight **(A)**, muscle weight **(B)**, fiber type composition **(C)**, and cross-sectional area of each muscle fiber type **(D)** were recorded. Data represent means ± SEM, *n* = 6 in each genotype. Means bearing different letters differ (*P* < 0.05).

Clenbuterol is a β2-adrenergic receptor agonist, and increases muscle mass and function (Shi et al., 2007; Kim et al., 2018; Ito et al., 2019). To test whether the adult muscle fiber in *Myh4*^–/–^ muscle responds to the growth promotant clenbuterol to the degree the wild-type mice fibers do, we fed the wild-type and *Myh4*^–/–^ mice 20 part per million clenbuterol in drinking water for 2 weeks. Clenbuterol significantly increased the body weight of the wild-type mice, whereas the body weight of the knockout mice remained unchanged (Figure 6A). Similarly, clenbuterol had a hypertrophic effect on muscle weight of gastrocnemius, whereas in *Myh4*^–/–^ muscle, such effect disappeared (Figure 6B). Of note, clenbuterol promoted I to IIA to IIX and to IIB conversion in wild-type muscle, whereas we saw a decrease in IIX fiber composition, though the proportion of type I and IIA fibers increased in *Myh4*^–/–^ muscle (Figure 6C). Accordingly, all the fiber types in the wild-type muscle increased significantly in size (Figure 6D). In contrast, IIX fibers in *Myh4*^–/–^ muscle decreased, though type I and IIA fibers increased in size (Figure 6D). Overall, these findings show clenbuterol cannot elicit a hypertrophic effect in mice lacking a functional *Myh4* gene compared to their wild-type counterparts and suggest the added lean growth accretion caused by beta-adrenergic agonist feeding may be requisite on changes in muscle fiber types, especially including type IIB fibers.

### Signaling Governing *Myh4* Gene Expression Remains Unchanged in *Myh4*^–/–^ Muscle

**FIGURE 7:**
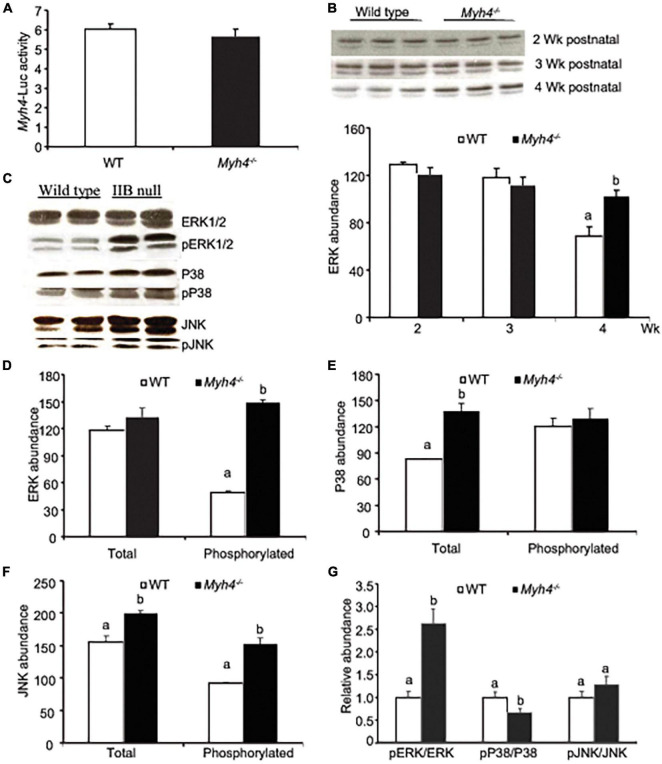
*Myh4* null mice retain normal fiber type signaling events compared to those in wild-type mice. **(A)** *Myh4*-Luc was electroporated into mouse *gastrocnemius* (GS) muscle and 1 week later lucifierase activity was assayed. **(B)** ERK MAPK signaling was assayed in GS muscle 2, 3, and 4 weeks postnatally. **(C–F)** ERK, p38, JNK and their phosphorylated forms were blotted **(C)** and quantified **(D–G)** in GS muscles from 4-week-old mice. Data represent means ± SEM, *n* = 6 males in each genotype. Means bearing different letters differ (*P* < 0.05). The phosphorylation sites of the MAPK antibodies are as follows: pERK1/2 (T202/Y204), pP38 (T180/Y182), and pJNK (T183/Y185).

To test whether the signals driving *Myh4* gene expression had changed in *Myh4*^–/–^ muscle, we electroporated a reporter gene carrying a *Myh4* promoter region, linked to luciferase into gastrocnemius (Figure 7A). The reporter gene activity remained unchanged in *Myh4*^–/–^ muscle, compared to the wild-type muscle (Figure 7A). Since the signaling of the MAPK family, ERK1/2 signaling in particular, is preferentially expressed in glycolytic muscles (Shi et al., 2008; Oishi et al., 2019), we measured the expression of ERK1/2 in wild-type and *Myh4*^–/–^ muscles postnatally. We observed that the protein level of ERK1/2 remained unchanged in weeks 2 and 3 postnatally, remaining at a high level at week 4 despite a decrease in its expression level at week 4 in wild-type muscle (Figure 7B). Furthermore, the phosphorylation level of ERK1/2 was significantly higher in *Myh4*^–/–^ muscle as compared to wild-type at week 4 (Figures 7C,D). Interestingly, the total levels of both p38 and JNK in *Myh4*^–/–^ muscle significantly increased compared to the wild-type, whereas only phosphorylated JNK increased in *Myh4*^–/–^ muscle (Figures 7C,E,F). To examine potential changes in signaling, we measured the ratio of all three phosphorylated MAPKs to their respective total MAPK proteins and found that pERK/ERK signaling significantly increased (Figure 7G). In contrast, pP38/P38 MAPK ratio decreased, whereas that of pJNK/JNK remained unchanged (Figure 7G). Together, these findings suggest that the signaling that drives the formation of fast-twitch fiber program remains unchanged even when *Myh4* gene is ablated.

## Discussion

In general, fast-twitch, glycolytic fibers account for 70–80% of muscle in mammals and vary in terms of developmental stage and the aging process. Not only are these fast-twitch fibers large in size, they also tend to experience the greatest hypertrophy postnatally in response to growth modulators, such as clenbuterol. In this study, we ablated *Myh4*^–/–^, and thus IIB fibers in mouse muscle, and investigated its role in muscle development and hypertrophy postnatally. We found (1) muscle fiber number remains unchanged until after birth, when IIB fibers progressively decreases between week 2 and 4; (2) muscle compensates for the loss of IIB fibers by increasing the proportion of type I and IIA fibers, and most notably, IIX fibers; (3) driving muscle metabolism from glycolytic to oxidative, either by genetically over-expressing PGC-1α or pharmaceutically by AICAR, partially rescues this loss in muscle mass and fiber number in *Myh4*^–/–^ muscle; (4) stimulating muscle growth in mice lacking *Myh4* either pharmacologically or through genetic approaches fails to rescue muscle mass and fiber number; and (5) those signal pathways driving fast-twitch program and IIB fiber formation remains intact in *Myh4*^–/–^ muscle.

Previous studies have shown that mice lacking *Myh4* have reduced muscle mass and fiber numbers (Acakpo-Satchivi et al., 1997; Allen and Leinwand, 2001), however, whether the loss of fiber number occurred before or after birth remained unknown. Herein, we show that fiber loss in *Myh4*^–/–^ muscle occurs after birth, specifically, between weeks 2 and 4 postnatally. This finding indicates that after birth, fetal muscle fiber types in mouse muscle are quickly replaced by more glycolytic, fast-twitch fibers to satisfy the needs for greater contractile power and speed. The disproportional change of muscle fiber composition favors the largest, most powerful, and fastest-twitch MyHC-IIB, and this event over a 2-week timeframe. Among all the driving forces for fiber type switching, we speculate that innervation may play a critical role in this process. There are three lines of evidence to support this argument: (1) cross-innervation can switch muscle fiber types to the opposite slow or fast phenotype (Buller et al., 1960; Hoh, 1975; Rhee and Hoh, 2010); (2) as many as a few dozen muscle fibers are innervated by a single motor axon to create a motor unit (Chakkalakal et al., 2010); and (3) a striking characteristic of this relationship is that all, or nearly all, muscle fibers, whether pure or hybrid, innervated by the same motor neuron are of the same fiber type, a phenomenon called motor unit homogeneity (Edstrom and Kugelberg, 1968; Burke et al., 1973; Burke, 1999). Based on these facts, we argue that motor neurons innervating IIB fibers may differ slightly from those controlling IIX fibers. As a result, fibers innervated by IIB-specific motor neurons may die or retrograde when fibers cannot express MyHC-IIB. That is why, despite an increase in IIX fibers in the gastrocnemius of *Myh4*^–/–^ mice, it cannot compensate for the loss of total muscle fibers. The argument that IIX and IIB fibers are not mutually replaceable postnatally is further supported by the fact that metabolic profiles and underlying signaling mechanisms in these two types are different. For example, in terms of speed of contraction, IIX fibers are intermediate to IIA and IIB fibers (Larsson et al., 1991; Bottinelli et al., 1994); yet in terms of metabolism, IIX fibers are more oxidative than IIB fibers, resembling type I and IIA fibers (Larsson et al., 1991). In a mouse knockout model, it has been shown that PGC-1β, a coactivator similar to PGC-1α, can drive the formation of IIX, but not IIB fibers, further arguing that IIX and IIB fibers have similar yet distinct identities (Arany et al., 2007). Alternatively, it is possible that the cell signaling program that gives rise to the MyHC-IIB also suppresses transcription all other MyHCs (Shi et al., 2007).

A major discovery in this study is that slowing the transition of muscle metabolism from oxidative to glycolytic phenotype, or specifically augmenting the signaling that promotes slow fiber type program, partially rescues muscle defects in *Myh4*^–/–^ mice. This finding argues that innervation may play a dominant role in specifying fiber type by defining myofibrillar calcium oscillation and downstream signaling events (Olson and Williams, 2000) required to match the metabolism of the cell with the contractile properties. On the other hand, shifting metabolism from oxidative to glycolytic phenotype, or vice versa, often leads to fiber type switching in the opposite direction (Wen et al., 2020; Xu et al., 2020a,b; Chen et al., 2021). For example, feeding mice with clenbuterol induces the *de novo* synthesis of IIX and IIB fibers in mouse soleus muscle, whose component are exclusively type I and IIA fiber (Shi et al., 2007). On the other hand, overexpression of FOXO1, a forked transcription factor enriched in fast-twitch muscle fibers, selectively downregulates slow-switch fibers (Kamei et al., 2004; Xu et al., 2017), potentially through its interaction with PGC-1α and MEF2 pathways (Xu et al., 2017). Our data show that forcing muscle metabolism to a more oxidative paradigm by PGC-1α overexpression only partially rescues muscle fiber loss in *Myh4*^–/–^ gastrocnemius. It is likely that the oxidative metabolism shifts IIB fibers to a IIX fiber-like phenotype, therefore saving IIB fibers from loss. Alternatively, overexpression of PGC-1α, which favors a slow fiber program, likely delays or blunts the slow-to-fast fiber transition program in a small proportion of fibers and keeps them viable longer. Of course, we cannot exclude the possibility that PGC-1α may change the firing pattern of the motor neuron innervating IIB fibers, since the overexpression is promiscuous, i.e., not restricted in skeletal muscle. Regardless, “pushing” the muscle phenotype to a more oxidative program contributes to the rescue of muscle mass and fiber number in *Myh4* null mice. This finding has significance in biomedicine. For example, sarcopenia is an age-related gradual loss of muscle fiber size, muscle mass, and muscle function (Rosenberg, 1997). A recent study using rat as the animal model reveals that 22-mo-old gastrocnemius muscle contains significantly less IIB fibers with a concomitant increase in the percentage of IIX fibers, as compared to its 9-mon-old adult counterpart (Faitg et al., 2019). This phenotype is similar to the *Myh4*^–/–^ gastrocnemius. Hence, we speculate that if we can switch the muscle metabolism to a more oxidative paradigm, we may ameliorate muscle wasting in aged people.

Another interesting observation is that muscle in in *Myh4*^–/–^ mice fails to respond to the hypertrophic actions either by myostatin knockout or by administration of the growth promotant clenbuterol. In other words, neither genetic nor pharmacological manipulations can fully compensate for the loss of MyHC IIB fibers in muscle. This phenomenon is in sharp contrast to that observed in lower-birth-weight lambs: the fiber number in low-birth-weight lambs are comparable to the normal lambs, and these lambs, whose body weights are as low as 38% of the normal lambs, compensate for this lowered body weight by 8 weeks postnatally (Greenwood et al., 1999, 2000; Louey et al., 2005). It appears the reduced muscle fiber number, particularly that of the largest IIB fibers, prevents full muscle hypertrophy postnatally. One of the reasons for the lack of response of the existing *Myh4*^–/–^ fibers to genetic and pharmacological manipulations can be that the compensatory enlargement of the IIX fibers in *Myh4*^–/–^ muscle cannot be further enlarged. In other words, IIX fiber has maximized its capacity to enlarge to compensate for the lack of its neighboring IIB fibers. Stimulating these fibers further cannot make them larger, and actually reduces number and cross-sectional area as in the case of clenbuterol feeding. In this regard, we propose that IIB fibers are required for maximal muscle mass accumulation and hypertrophy during muscle growth postnatally. Still, it remains an enigma why IIX fibers would experience negative growth in an environment where hypertrophic signals presumably dominate unless, as mentioned above stimulation for one program (IIB) is also accompanied with repressing signal of the other program (IIX) as proposed by Shi et al. (2007).

In summary, our findings reveal MyHC IIB plays a key role in postnatal muscle growth and development in mice, and promoting an oxidative phenotype can prevent muscle fiber loss in mice lacking type IIB fibers. These findings not only aid our understanding of the transition of fiber type postnatally, but also provide potential directions for medical intervention to treat debilitating muscle diseases such as sarcopenia, atrophy and muscular dystrophy.

## Data Availability Statement

The original contributions presented in the study are included in the article/supplementary material, further inquiries can be directed to the corresponding author/s.

## Ethics Statement

The animal study was reviewed and approved by the Purdue Animal Use and Care Committee.

## Author Contributions

CZ, HS, KH, AG, and DG contributed to this research and subsequent manuscript from conception to final preparation. LK, AR, SP, and JS helped design, collect, and analyze data. All authors contributed to the article and approved the submitted version.

## Conflict of Interest

The authors declare that the research was conducted in the absence of any commercial or financial relationships that could be construed as a potential conflict of interest.

## Publisher’s Note

All claims expressed in this article are solely those of the authors and do not necessarily represent those of their affiliated organizations, or those of the publisher, the editors and the reviewers. Any product that may be evaluated in this article, or claim that may be made by its manufacturer, is not guaranteed or endorsed by the publisher.
